# Low-density lipoprotein cholesterol estimation by the Anandaraja's formula – confirmation

**DOI:** 10.1186/1476-511X-5-18

**Published:** 2006-06-29

**Authors:** Rudolf Gasko

**Affiliations:** 1 LABMED, a.s., clinical laboratory, University Hospital L. Pasteur, Rastislavova 43, SK-04001 Kosice, Slovak Republic

## Abstract

The number of the indirect methods for LDL-C estimation is growing. Our result support the reliability of new Anandaraja's formula for low-density lipoprotein estimation from total cholesterol and triglycerides.

Gazi et al [1] presented results of evaluation the accuracy of various formulas for the calculation of low-density lipoprotein cholesterol (LDL-C) levels – indirect methods – in patients with the metabolic syndrome. LDL-C according Friedewald's formula showed significant differences from other formulas in the total cohort, as well as in patients with metabolic syndrome. Different plasma lipid and lipoprotein analytical techniques – direct methods, however, yield results, which are highly correlated, yet significantly different, too [2].

It is possible to supplement next calculation. Anandaraja et al [3] published new formula for LDL-C estimation from two other parameters, total cholesterol and triglycerides, as substitution to well known Friedewald's formula.

Our team recently compared two methods of estimation of low-density lipoprotein cholesterol, the direct Wako method versus Friedewald's formula [4]. We studied over 10 000 consecutive patients with different diseases, from mixed rural and urban Brazil population.

We re-counted our data with Anandaraja's formula. Main results are expressed in Table 1. Direct LDL-C and LDL-C calculated from Anandaraja's formula shows substantial better correlation in both total population (r = 0,969) and patients with triglyceride <350 mg/dl (r = 0,974) by comparison with calculated according to the Friedewald's formula (r = 0,924 and r = 0,935).

**Table 1:** Low-density lipoprotein cholesterol level

Particulars	Total population	Patients with triglycerides <350 mg/dl (3,94 mmol/l)
Total sample number	10 324	10 102
Direct low-density lipoprotein cholesterol (mg/dl; mmol/l)	116 ± 22 (2,99 ± 0,57)	115 ± 22 (2,97 ± 0,57)
Calculated low-density lipoprotein cholesterol (mg/dl; mmol/l)*	120 ± 24 (3,10 ± 0,62)	119 ± 23 (3,08 ± 0,59)
Calculated low-density lipoprotein cholesterol (mg/dl; mmol/l)†	115 ± 23 (2,97 ± 0,59)	114 ± 21 (2,95 ± 0,54)
Difference between direct low-density lipoprotein cholesterol and calculated low-density lipoprotein cholesterol (mg/dl)*	- 4 ± 16	- 4 ± 14
Difference between direct low-density lipoprotein cholesterol and calculated low-density lipoprotein cholesterol (mg/dl) †	1 ± 14	1 ± 14
Correlation coefficient between direct low-density lipoprotein cholesterol and calculated low-density lipoprotein cholesterol*	0,924	0,935
Correlation coefficient between direct low-density lipoprotein cholesterol and calculated low-density lipoprotein cholesterol†	0,969	0,974

All measurements are expressed in mg/dl (mmol/l) and results are presented as mean ± S.D. Pearson's correlation coefficient used. Direct LDL-C measured with Wako method – for details see Ref. [4].

* Calculated low-density lipoprotein cholesterol from Friedewald's formula

† Calculated low-density lipoprotein cholesterol from new Anandaraja's equation

In each very low-density lipoprotein particle secreted from the liver, and therefore in each intermediate-density lipoprotein, low-density lipoprotein and lipoprotein (a) particles, a single molecule of apolipoprotein B (apo B) is present from the time of its assembly to its catabolism, which does not exchange between lipoproteins. More than 90% of apo B is in the low-density lipoprotein particles, and therefore, plasma total concentration of apo B can be considered as a measure of the number of atherogenic particles. New Anandaraja's equation takes into account only total cholesterol and triglycerides. Some authors recommend including apo B measurement as acomponent of the standard lipoprotein measurements performed to assess cardiovascular disease risk [5]. Contribution of LDL-C calculation with help of measured apo B is questionable. Apo B is real an independent risk factor.

In conclusion, LDL-C is the major indicator for initial classification of coronary heart disease risk status and lowering its level is a primary goal of therapy. The NCEP Working Group on Lipoprotein Measurement recommended the development of accurate direct low-density lipoprotein cholesterol methods. All available direct methods have yet limitations for general use. Our results support the reliability of Anandaraja's formula as indirect low-density lipoprotein cholesterol estimation – originally used in Indian population – in Brazil population, too.
